# CAREGIVING APPROACHES TO RESISTIVE BEHAVIORS AMONG PERSONS LIVING WITH DEMENTIA IN LONG-TERM CARE

**DOI:** 10.1093/geroni/igad104.2285

**Published:** 2023-12-21

**Authors:** Kyung Hee Lee, Ji Yeon Lee, Ji Yeong Park

**Affiliations:** Yonsei University, Seoul, Republic of Korea; Yonsei University, Seoul, Republic of Korea; Yonsei University, Seoul, Republic of Korea

## Abstract

Many older adults living in long-term care have dementia sufficiently severe to interfere with the performance of basic activities of daily living; this number is expected to increase rapidly. Although resistive behaviors are common among long-term care residents with dementia during care, caregivers face difficulty to prevent and manage them. The purpose of this study was to examine how caregivers responded to resistiveness to care of persons living with dementia. We analyzed 107 videos from 24 residents with dementia and 34 caregivers in four long-term care facilities. Each interaction between resident with dementia and caregiver was taken nine times at 0, 3, and 6 months at three care events (i.e., personal care, mealtime, and activity). Resistiveness to care was assessed using the Resistiveness to Care Scale for Dementia of the Alzheimer’s Type; caregiving approaches were coded by the Person-Centered Behavior Inventory and the Task-Centered Behavior Inventory. Lag sequential analysis was conducted to examine caregiving approaches right after resistive behaviors of residents with dementia. We analyzed 25.1 hours of care events; 544 episodes of resistiveness to care were observed. When residents with dementia showed resistiveness to care, caregivers provided person-centered behaviors in 34.4% of cases, task-centered behaviors in 35.8% of cases, and nothing in 29.8% of cases. Caregivers used task-centered care approaches or nothing to manage resistiveness to care in more than 65% of cases. It is recommended to provide person-centered caregiving approaches to deal with resistiveness to care among persons living with dementia.

